# TIMP1 mRNA in tumor-educated platelets is diagnostic biomarker for colorectal cancer

**DOI:** 10.18632/aging.102366

**Published:** 2019-10-22

**Authors:** Liu Yang, Qian Jiang, Dong-Zheng Li, Xin Zhou, Dong-Sheng Yu, Jian Zhong

**Affiliations:** 1 Department of Colorectal Surgery, Jiangsu Cancer Hospital and Jiangsu Institute of Cancer Research and The Affiliated Cancer Hospital of Nanjing Medical University, Nanjing, China

**Keywords:** platelet, RNA, biomarker, colorectal cancer, TIMP1

## Abstract

Platelets have been shown to promote the growth of tumors, including colorectal cancer. The RNA profile of tumor-educated platelets has the possibility for cancer diagnosis. We used RNA sequencing to identified the gene expression signature in platelets from colorectal cancer patients and healthy volunteers. We then verified the selected biomarkers from the RNA sequencing in a two-step case-control study using quantitative reverse-transcription polymerase chain reaction. We found that TIMP1 mRNA levels are higher in platelets from colorectal cancer patients than in platelets from healthy volunteers and patients with inflammatory bowel diseases. Additionally, TIMP1 mRNA expressed in platelets from colorectal cancer patients can be carried into colorectal cancer cells, where it promotes tumor growth *in vivo* and *in vitro*. These findings show that the TIMP1 mRNA in platelets is a potential independent diagnostic biomarker for colorectal cancer, and that platelets can carry RNAs into colorectal cancer cells to promote colorectal cancer development.

## INTRODUCTION

Colorectal cancer (CRC) is a very malignant tumor with high morbidity and mortality rates [1]. Most CRC patients are already in advanced stages when diagnosed because the early stages have no symptoms. The morbidity and mortality of CRC can be reduced by proper screening methods, which can detect precancerous lesions and early cancers. Colonoscopy is the first option recommended by CRC screening guidelines. However, colonoscopy involves high costs, significant resources, discomfort from bowel preparation, and a higher risk of complications.

The blood-based liquid biopsy to detect free DNA (ctDNA) or circulating tumor cells has been shown to be a possible noninvasive method for CRC screening [2]. However, this technique requires a complicated separation processes and cannot be used for early diagnosis because the amounts of ctDNA and CTCs are too low [2–3]. Platelets are multifunctional non-nuclear cell fragments originating from bone marrow megakaryocytes, which are abundant in peripheral blood. Platelets have been confirmed to promote tumor development and metastasis [4–5]. The RNAs and proteins in platelets can be directly or indirectly promoted by tumor cells and the tumor microenvironment, leading to the formation of tumor-educated platelets (TEPs), which, in turn, promote cancer progression. Compared with platelets from healthy control subjects, TEPs from tumor patients are phenotypically and functionally different, which raises the possibility that they could serve as a tumor marker [6–7]. For example, Peterson et al. [8] found that levels of VEGF, PDGF, and PF4 are higher in the platelets from CRC patients than in age-matched healthy control subjects. In addition, Best et al. [9–10] showed that the spliced RNA profile within platelets is altered in cancer. Platelet RNA from patients with different cancers or healthy control subjects was sequenced and analyzed using an algorithm to select an RNA biomarker panel that can be used to differentiate patients from control subjects. The platelet RNA panel was validated in an independent cohort, and was then used to accurately differentiate early-stage and late-stage cancer patients from healthy control subjects [9–10].

Nilsson et al. [11–14] observed that platelets from patients with glioblastoma, prostate cancer, and non-small cell lung cancer (NSCLC) sequester tumor-derived mutant RNA molecules, suggesting [11–14] RNA in platelets could complement currently used biosources and biomolecules in liquid biopsy diagnosis of cancer. In the present study, we characterized the platelet RNA profiles of CRC patients and healthy donors and investigated their potential for TEP-based CRC diagnostics. We also explored the activity of increased RNA in platelets from CRC patients in the development of CRC

## RESULTS

### Weighted correlation network analysis

**Figure 1:**
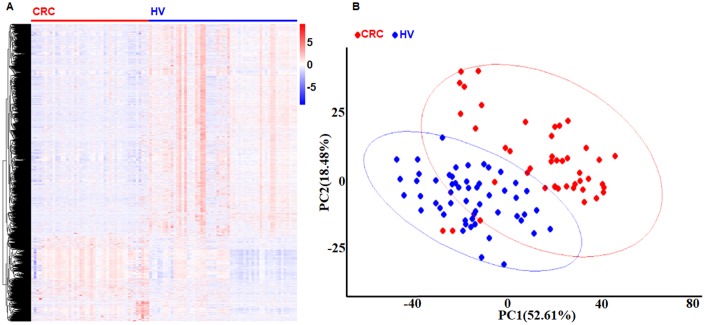
**Identification DEGs in platelets from CRC and HVs.** (**A**) The heat-map of gene expression profiles in platelets from CRC and HVs. Red indicates a higher expression and green indicates a lower expression. (**B**) The PCA plot of gene expression profiles in platelets from CRC and HVs. Red bar: CRC group. Blue bar: HV group.

**Figure 2:**
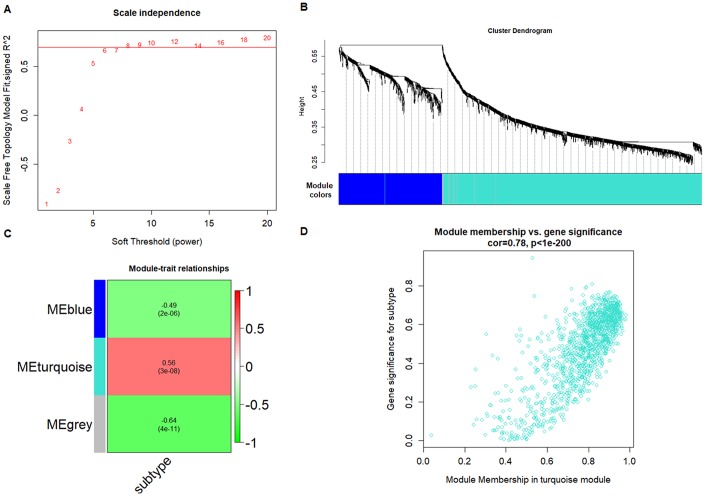
**WGCNA screening of platelet RNAs in CRC patients.** (**A**) Analysis of network topology for various soft-thresholding powers. (**B**) Clustering dendrograms of genes, with dissimilarity based on topological overlap, together with assigned module colors. (**C**) Module-trait associations. Each row corresponds to a module eigengene, column to a trait. Each cell contains the corresponding correlation and *P*-value. The table is color-coded by correlation according to the color legend. (**D**) A scatterplot of Gene Significance (GS) for subtype vs. Module Membership (MM) in the turquoise module.

We used 99 platelet samples, including 44 from CRC patients and 55 from healthy volunteers (HVs) to perform the co-expression network by Weighted correlation network analysis (WGCNA) in the R software package. First, we found 1099 different expression genes (DEGs) in the platelets, including 824 increased DEGs and 275 decreased DEGs between the CRC group and the HV group with *P* < 0.001 and fold change > 1 by limma in R software package. As showed in Figure 1, both the heatmap and the principle component analysis (PCA) plot showed the DEGs can distinguish the CRC samples from the HV samples. Second, the power 1 was chosen as the soft-threshold (Figure 2A), in which the connections between the genes in the network were close to the scale-free network (Supplementary Figure 1). Third, we constructed the co-expression modules and identified three distinct modules (Figure 2B). Three modules, including turquoise (1296 DEGs), blue (524 DEGs), and gray (25 DEGs) were obtained (Figure 2B). Because the DEGs in the gray module were not included in any other module, the gray module was not used in subsequent analyses. ME (moduleEigengenes) was in accordance with the expression pattern of DEGs in each module. The turquoise module was increased and positively correlated with the disease (correlation index: 0.56, *P* = 3.0E–08) (Figure 2C). The GS (gene significance) value for turquoise module was 0.78, which indicated a close correlation with the disease (Figure 2D). According to the network topological index, 10 hub genes (ASAH1, C12orf76, FRMD3, SVIP, ADIPOR1, TIMP1, RAB4A, ISCU, PGRMC1, and CALM3) were investigated from the turquoise modules (Table 1).

**Table 1:** The 10 hub genes.

**Gene symbol**	**FC**	**Ave. Expr.**	***t***	***P*. value**	**adj. *P*. value**	**B**
ASAH1	5.418667	11.33776	10.12793	1.69E-18	1.69E-17	31.37766
C12orf76	4.290505	5.969176	9.127999	6.14E-16	3.00E-15	25.54862
FRMD3	4.068121	8.089412	9.062479	8.99E-16	3.00E-15	25.17152
SVIP	3.325292	9.457059	8.915989	2.10E-15	5.26E-15	24.33104
ADIPOR1	4.088759	10.77271	8.758637	5.22E-15	1.04E-14	23.43254
TIMP1	4.103427	11.25247	8.685446	7.96E-15	1.33E-14	23.01621
RAB4A	3.868231	9.100941	8.497088	2.34E-14	3.35E-14	21.94975
ISCU	3.255445	8.867059	8.255502	9.27E-14	1.15E-13	20.59314
PGRMC1	3.309016	10.04024	8.236421	1.03E-13	1.15E-13	20.48656

### TIMP1 increased in the platelets from CRC patients

**Figure 3:**
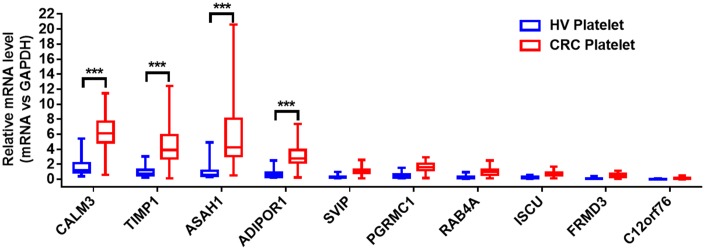
The relative levels of 10 hub genes in platelets from 20 CRC patients and 20 HVs by qRT- PCR. ***P < 0.001.

**Figure 4:**
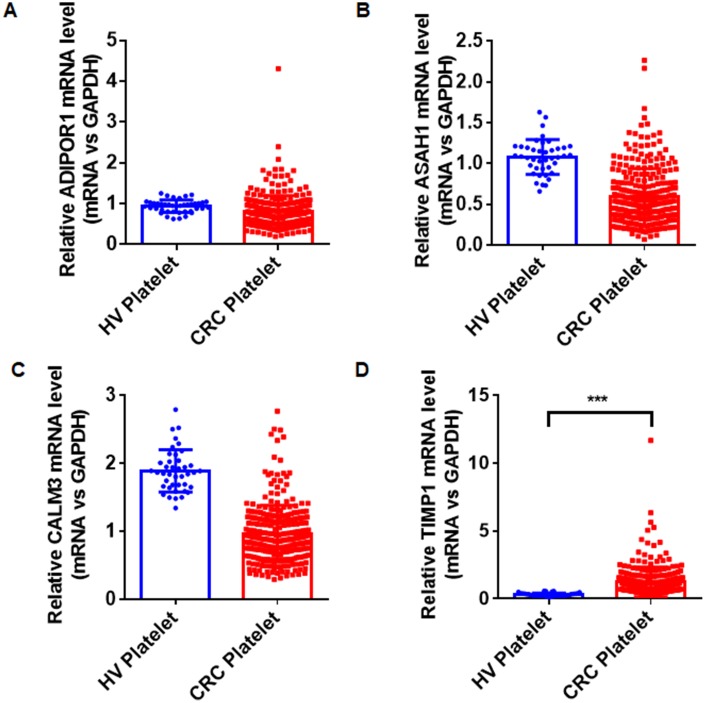
The relative levels of ADIPOR1 (**A**), ASAH1 (**B**), CALM3 (**C**), and TIMP1 (**D**) mRNAs in the platelets from 286 CRC patients and 41 HVs by qRT- PCR. ***P < 0.001.

The quantitative reverse transcription polymerase chain reaction (qRT-PCR) assay was used to validate the selected hub genes from the WGCNA in two independent cohorts. We prospectively collected and isolated platelets from 1-mL blood and confirmed the platelet purity by morphological analysis (contamination is 1 to 5 nucleated cells per 10 million platelets). Subsequently, we isolated the platelet RNA by use of the RNeasy Mini Kit (Qiagen, Germany) and evaluated it by use of Nanodrop (ThermoFisher). We isolated 2 ng–10 ng RNA from the platelets of 1 mL blood. The characteristics of the patients and healthy control subjects enrolled in the training and validation sets are given in Table 2. In the training set, we examined the mRNAs of platelets from 20 CRC patients and 20 HVs. The mRNAs with fold change > 2, and *P* < 0.01were selected. The four stimulated mRNAs (CALM3, TIMP1, ASAH1, and ADIPOR1) were significantly increased (Figure 3) and chosen for further validation in a larger cohort (validation set) involving 286 CRC patients and 41 matched HVs. As shown in Figure 4, the TIMP1 mRNA levels were higher in platelets from CRC patients than in platelets from healthy individuals (Figure 4D). The other three mRNAs show no difference (Figure 4A–4C). The TIMP1 mRNA in the platelets gradually slightly increased with the development of CRC (Supplementary Figure 2A). More importantly, The TIMP1 mRNA in the platelets significantly elevated in the late stage (stage III/IV) compared to the early stage (stage I/II) (Supplementary Figure 2B). Additionally, we also investigated the expression of TIMP1 mRNA in the platelets from 22 patients with ulcerative colitis and 23 patients with Crohn’s disease. As showed in Supplementary Figure 3, the TIMP1 mRNA levels were higher in the platelets from CRC patients, compared with the platelets from patients with ulcerative colitis or Crohn’s disease.

**Table 2:** Patient characteristics and clinical features.

		**N**	**CRC**	**Ulcerative colitis**	**Crohn’s disease**
Age		70.6±12.6	67.4±12.9	68.2±8.6	61.9±11.2
Sex					
	Female	21	151	12	12
	Male	20	135	10	11
Type					
	Colon Mucinous Adenocarcinoma		42		
	Colon Adenocarcinoma		244		
Stage					
	I		66		
	II		162		
	III		23		
	IV		35		
T					
	1		10		
	2		55		
	3		194		
	4		27		
N					
	0		232		
	1		23		
	2		31		
M					
	0		221		
	1		65		

### Receiver operating characteristic curve analysis

**Figure 5:**
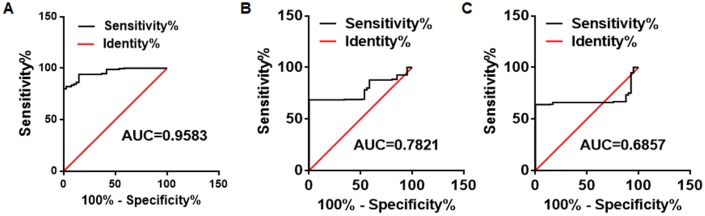
**The ROC analysis for differentiation ability between CRC patients and healthy control subjects.** ROC analyses for the TIMP1 mRNAs in the platelets, CEA, and CA199 to differentiate 286 CRC patients and 41 HVs.

We used receiver operating characteristic (ROC) curves to analyze the availability of the TIMP1 mRNA from platelets in the differential diagnosis of CRC patients and HVs. The analysis showed the area under the curve (AUC) for the ROC curve for TIMP1 mRNA was 0.9583 (95% CI, 0.9363–0.9803) (Figure 5A). Because the carcinoembryonic antigen (CEA) and carbohydrate antigen 199 (CA199) assays are the most commonly used tests for colon cancer, we also analyzed the ROC curves of CEA and CA199 in the CRC patients. Consistent with previous reports, the AUC for CEA and CA199 were approximately 0.7654 and 0.6123 (Figure 5B–5C). These results suggest that the detection of TIMP1 mRNA in the platelets has higher sensitivity and specificity than the detection of CEA or CA199 for CRC diagnosis.

### TIPM1 mRNA carried into cancer cells by tumor-educated platelets promotes cancer cell growth

**Figure 6:**
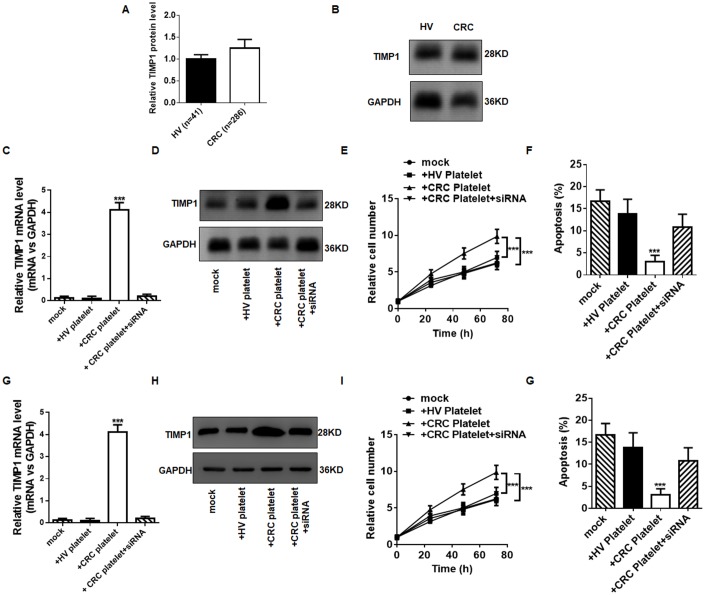
**TEPs promote CRC cell proliferation and inhibit cancer cell apoptosis.** (**A**–**B**) The protein level of TIMP1 in platelets from HV and CRC by ELISA (**A**) and western blotting (**B**). (**C**–**D**) The mRNA (**C**) and protein (**D**) level in HT29 cells incubated with platelets from CRC patients and HVs. (**E**) The proliferation of HT29 cells exposed to platelets from HVs and colorectal cancer patients were determined by CCK-8 assays. (**F**) Apoptosis of HT29 cells exposed to platelets from healthy volunteers and colon cancer patients for 72 h were determined by flow cytometry. (**G**–**H**) The mRNA (**G**) and protein (**H**) level in Caco-2 cells incubated with platelets from CRC patients and HVs. (**I**) The proliferation of Caco-2 cells exposed to platelets from HVs and colon cancer patients were determined by CCK-8 assays. (**G**) Apoptosis of Caco-2 cells exposed to platelets from HVs and colon cancer patients for 72 h were determined by flow cytometry. **P < 0.01, ***P < 0.001.

Evidence suggests that tumor cells can activate platelets in multiple ways, resulting in TEPs, and activated platelets can, in turn, promote tumor growth and metastasis. Our results show that the RNA profile in the platelets from CRC patients are different the RNA profile in the platelets from HVs, especially the levels of TIMP1 mRNA. To reveal the activity of TIMP1 mRNA in the TEPs during CRC progression, we investigated the protein level of TIMP1 in the platelets from HVs and CRC patients. The protein level of TIMP1 in the platelets was not different in HVs and CRC patients (Figure 6A and 6B), although the mRNA level of TIMP1 increased (Figure 4D). We considered whether platelets can carry TIMP1 mRNA to CRC cells. CRC cell lines HT29 and Caco-2 were incubated with the platelets from HVs and CRC patients. Incubation of HT29 cells or Caco-2 cells with platelets from CRC patients strongly increased TIMP1 mRNA level, and this elevation can be suppressed by co-transfecting HT29 cells with TIMP1 siRNA (Figure 6C and 6G). In contrast, the pre-TIMP1 mRNA level (primer: Forward, TTCTGGCATCCTGTTGTTGC; reverse, CACTGGACTGAAAGGGAAACCA) in the recipient HT29 cells was not altered by incubation with platelets (Supplementary Figure 4A and 4B), suggesting that the increase of TIMP1 mRNA level in cancer cells is not caused de novo mRNA biosynthesis but derived from platelet delivery. To determine whether exogenous TIMP1 mRNA delivered by platelet has a biological function in cancer cells, we analyzed the TIMP1 protein level in HT29 cells and Caco-2 cells. As shown in Figure 6D and 6H, incubation of cancer cells with platelets from CRC patients increased the levels of cellular TIMP1 protein, and this increase of TIMP1 protein was suppressed by co-transfection with the TIMP1 siRNA. These results confirmed the activity of TIMP1 mRNA carried by platelets in increasing TIMP1 levels in the cancer cells. The platelets of CRC patients increased the TIMP1 protein level in cancer cells compared with platelets of HVs (Figure 6D and 6H). TIMP1 encodes a 931 base-pair mRNA and a 207 amino acid soluble and widely distributed protein. This protein is crucial for the tumor progression because it promotes cell proliferation and apoptosis. We determined the possible activity of TIMP1 mRNA carried by platelets in stimulating cancer cells proliferation and apoptosis. To reduce TIMP1, a siRNA that inhibits TIMP1 was designed and then transfected into HT29 cells. Efficient reduction of TIMP1 in HT29 cells is shown in (Supplementary Figure 4C and 4D). HT29 cells transfected with TIMP1 siRNA showed a decreased proliferation and decreased apoptosis (Supplementary Figure 4E and 4F). Compared with cells incubated with platelets from HVs, those incubated with platelets from CRC patients exhibited higher proliferation rates (Figure 6E and 6I) and lower apoptosis rates (Figure 6F and 6G), and this promotion of proliferation and inhibition of apoptosis was attenuated by co-transfecting HT29 cells with TIMP1 siRNA. These results show that platelet-delivered TIMP1 mRNA can be carried into cancer cells, in which it can be translated to proteins and thus promote cell proliferation and inhibit cell apoptosis.

### TEPs promote CRC progression

**Figure 7:**
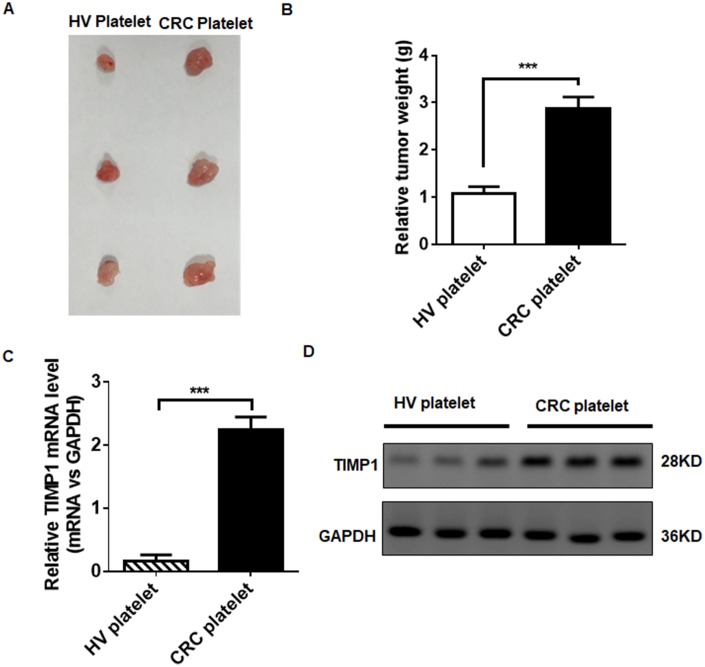
**TEPs promote CRC growth.** (**A**–**B**) HT29 tumor-bearing mice were excised at 24 days, imaged macroscopically, and weighed. (**C**–**D**) The mRNA © and protein (**D**) levels in tumors injected with platelets from CRC patients and HVs. **P < 0.01, ***P < 0.01.

We tested whether platelets can promote the tumorigenicity of CRC in vivo using a xenograft model in nude mice by injecting HT29 cells into the left side of the armpit. As shown in Figure 7A, the tumors injected with platelets from CRC patients were significantly larger than the tumors injected with platelets from HVs. Tumor weight was increased in CRC patients’ platelets injected tumors (Figure 7B), indicating that TEPs have oncogenic activity during colorectal progression. As with the results in vitro, the TIMP1 mRNA and protein increased in tumors injected with platelets from CRC patients (Figure 7C and 7D). These data suggest that platelets from CRC patients are active in CRC growth.

## DISCUSSION

Platelets have long been thought to just stimulate coagulation after tissue trauma or vascular injury. However, it is now known that platelets promote tumor growth and metastasis through secretion of proteins and RNAs that accelerate tumor proliferation [15–16].

Although the number of RNAs in platelets is low, studies suggest platelets have a rich mRNA repertoire (approximately 3000 to 6000 mRNAs per platelet). Several investigations have found that platelets can stimulate the splicing of their pre-mRNAs in response to signals from cancer cells, resulting in changes in their transcriptome profiles that can reflect pathological progression. Therefore, platelet mRNA can be exploited for use in liquid biopsies. Best et al. [9] performed RNA sequencing on 283 blood platelet samples isolated from healthy individuals (n = 55) and patients with cancer (n = 228; cancer types: glioblastoma, NSCLC, CRC, pancreatic cancer, breast cancer, and liver and bile duct carcinoma) [9]. The unsupervised hierarchical clustering of two sample groups (cancer patients vs. healthy volunteers) has only minor overlap based on the differentially detected platelet mRNAs. Those investigators developed a vector machine (SVM) classification using the different mRNA profiles of cancer patients and healthy donors that enabled accurate separation of healthy individuals from cancer patients (accuracy: 96%). In a follow-up study [10], they used article-swarm optimization (PSO)-enhanced algorithms to select RNA biomarker panels from RNA sequencing libraries of platelets from NSCLC patients [10]. The platelet RNA panel was subsequently validated in an independent cohort, which enabled highly accurate differentiation of early-stage and late-stage cancer patients from healthy control subjects. Studies have shown that platelets can absorb tumor-derived RNAs contained in microvesicles secreted by tumor cells. Nilsson et al. [11, 12] described how platelets can sequester extracellular vesicles (EVs) from cancer cells harboring tumor-specific RNA, and a specific tumor RNA, EGFRvIII (a deletion mutant of the epidermal growth factor receptor [EGFR]), can be detected in platelets from cancer patients. Using RT-PCR and deep amplicon sequencing, Best et al. [9] also detected tumor-derived RNA, including translocated EML4-ALK transcripts as well as KRAS and EGFR transcripts harboring tumor-specific point mutations, in platelets from NSCLC patients. Overall, these studies suggest that TEP RNAs are potentially useful biomarkers for disease diagnosis, prognosis, and prediction.

Compared with the resources currently used for liquid biopsy, TEP RNAs have several advantages as biomarkers for cancer diagnosis. There is no interference from genomic DNA because platelets contain no nuclei. Platelets are easy to collect, isolate and analyze. Platelets are continuously exposed to the tumor and its environment, exchanging biomolecules with the tumor cells, resulting in TEP formation. Analysis of platelet RNA profiles or direct measurement of tumor-derived biomarkers within platelets provide information on cancer-related processes in the individual (e.g., early diagnosis for cancer, oncogenic mutation for treatment selection).

In our study, the platelet RNA profiles of CRC patients and healthy donors were screened for potential biomarkers for CRC diagnosis. Use of the WGCNA package in R, we analyzed 1099 DEGs, including 824 stimulated DEGs and 275 suppressed DEGs, and found 10 hub genes, including ASAH1, C12orf76, FRMD3, SVIP, ADIPOR1, TIMP1, RAB4A, ISCU, PGRMC1, and CALM3. Our qRT-PCR assays verify the 10 hub genes in a small cohort, including 20 CRC patients and 20 HVs. The four stimulated mRNAs (CALM3, TIMP1, ASAH1, and ADIPOR1) with fold changes >2 and *P* < 0.01 were selected for further validation in a larger cohort (validation set) of 286 CRC patients and 41 matched HVs. As the result showed in Figure 4, only one biomarker out of the 10 hits in the hub was validated. TIMP1 mRNA was increased in platelets from cancer patients, but there was no difference in the other three RNAs. This might need perform more sequencing and qRT-PCR assays in a much larger group to find the potential factors. We further used ROC curves to analyze the availability of the TIMP1 mRNA from platelets in the differential diagnosis of CRC patients and HVs. The ROC curve for TIMP1 mRNA in platelets was 0.9583 (95%CI, 0.9363–0.9803), which is much higher than for CEA and CA199, which are the most commonly used tests for CRC diagnosis.

TIMP1 is located on chromosome Xp11.3-p11.23. Its transcript is a 931 base-pair mRNA encoding a 207 amino acid protein. This protein inhibits the proteolytic activity of matrix metalloproteinases (MMPs) and disrupts the balance of matrix remodeling during degradation of extracellular matrix, which are crucial for tumor invasion and metastasis. TIMP1 also stimulates cancer cell proliferation and apoptosis. Batra et al demonstrated that overexpression of TIMP1 can increase expression of genes during proliferation and apoptosis [17]. In addition, TIMP1 reportedly degrades cyclinB1, activates the NF-kβ signaling pathway, and binds to the CD63/integrin β1 complex, causing anti-apoptotic effects. Song et al reported that TIMP1 activates the FAK-PI3K/AKT and the MAPK pathways to promote CRC progression and metastasis [18]. We found that TIMP1 mRNA was increased in platelets from CRC. However, there was no difference in the TIMP1 protein levels between platelets from cancer patients and HVs. TIMP1 mRNA is nearly absent in platelets from healthy humans, but is increased in platelets from tumor patients. This difference suggests that TIMP1 mRNA is likely to be induced by cancer cells during platelet maturation or absorbed by platelets from tumor cells

In conclusion, we have shown that TIMP1 mRNA in platelets could potentially serve as a non-invasive biomarker for diagnosing CRC.

## MATERIALS AND METHODS

### Patient characteristics

Blood samples from 286 patients with the newly diagnosed untreated CRC were included in this study. The control subjects were 41 healthy age-matched volunteers, 22 patients with ulcerative colitis and 23 patients with Crohn’s disease. All the patients were diagnosed at the Department of Colorectal Surgery, Jiangsu Cancer Hospital and Jiangsu Institute of Cancer Research and The Affiliated Cancer Hospital of Nanjing Medical University. The clinicopathological data of all CRC patients were collected. This study was approved by the Ethics Committee of Jiangsu Institute of Cancer Research, and written informed consent was obtained from all patients. All experiments were performed in accordance with relevant guidelines and regulations.

### Platelet gene-expression profiles of CRC

Platelet gene-expression profiles of CRC patients were downloaded from the Gene Expression Omnibus with accession number GSE68086. We used 44 platelet samples (CRC group) obtained from CRC patients and 55 platelet samples from HVs in the dataset (GSE68086) for the follow-up analysis. The dataset was normalized by the limma package, and the DEGs between the CRC group and the HV group were subsequently analyzed by the eBayes function in limma. WGCNA was performed to analyze the co-expression network and the hub genes, which could be potential biomarkers in the platelets for the CRC diagnosis.

### Platelet isolation and RNA extraction

To isolate platelets, whole blood was centrifuged at 300 × g for 30 min. to separated platelet-rich plasma from nucleated blood cells. The platelet-rich plasma was diluted in washing buffer (10 mM HEPES, 136 mM NaCl, 2.7 mM KCl, 2 mM MgCl2, 25 mM glucose, 4.2 mM EDTA, 4.2 mM trisodium citrate, and 1 mM PGE, pH 6.6) and centrifuged at 800 g for 20 min. Subsequently, the pellet was re-suspended in washing buffer without PGE (prostaglandin E) and centrifuged in the same conditions. To assess platelet purity, freshly isolated and randomly selected platelet isolations were fixed in 3.7% paraformaldehyde and stained by Crystal-Violet staining (ratio 1:1). Total platelet and nucleated cell counts was determined by manual cell counting in 7 μL cell counting chambers on a light microscope. Platelet pellets were frozen at −80° C. For platelet RNA isolation, frozen platelets were thawed on ice, and total RNA was isolated using the RNeasy Mini Kit (Qiagen, Germany) according the manuscript.

### Quantitative real-time reverse transcription polymerase chain reaction

qRT-PCR performed with a CFX Connect Real-Time System (Bio-Rad Laboratories, Hercules, CA, USA) using the TaqMan® Reverse Transcription Kit and the TaqMan® Fast Advanced Master Mix (Applied Biosystems, Foster City, CA, USA) according the manufacturer’s instructions. The primers used in the study were given in Supplementary Table 1.

### Cell culture

HT29 and Caco-2 cells were purchased from the Institute of Cell Biology at the Chinese Academy of Sciences (Shanghai, China) and cultured in DMEM supplemented with 10% FBS and 1% penicillin/streptomycin in a humidified atmosphere containing 5% CO_2_/95% air at 37° C.

### Treatment of CRC cells with platelets for in vitro assay

HT29 or Caco-2 cells were seeded in the corresponding medium with 10% FBS. Immediately before treatment, the medium was changed for fresh medium. A total of 1×10^8^/mL platelets from CRC patients or HVs were incubated with HT29 or Caco-2 cells overnight.

### Enzyme-linked immunosorbent assay and western blotting

For enzyme-linked immunosorbent assay (ELISA), platelets were homogenized in lysis buffer containing 10 mm Tris-HCl, pH 7.4, 150 mm NaCl, 1 mm EDTA, 0.5% Triton X-100 and protease inhibitor cocktail (Sigma). Total levels of TIMP-1 were measured in lysates following the protocol for the Human TIMP-1 ELISA Kit (RAB0467, Millipore). For western blotting, the collected cells were homogenized in RIPA buffer (50 mm Tris-HCl, pH 7.4, 150 mm NaCl, 1 mm EDTA, pH 8.0, 1% Triton X-100, 0.1% SDS, and 0.5% 21-hydroxyprogesterone) containing a protease inhibitor cocktail (Sigma) and 0.5 mm sodium pervanadate. Supernatants were isolated and resolved by SDS-PAGE with reducing sample buffer before being transferred onto the appropriate membrane and incubated with antibodies. Primary antibodies for TIMP1 (WH0007076M1, Sigma-Aldrich) and GAPDH (G8795, Sigma-Aldrich), and secondary antibodies anti-mouse-HRP (A9044, Sigma-Aldrich) were used for detection. Band intensities were quantified using ImageJ software, and the results were normalized to GAPDH and expressed relative to control subjects.

### Cell proliferation assay

Cell Counting Kit-8 (CCK-8) assays were performed to measure the proliferation rate of HT29 or Caco-2 incubated with platelets. HT29 or Caco-2 incubated with platelets was seeded in 24-well plates. For the CCK-8 assay, Cell Counting Kit-8 (Dojindo, Japan) was added into cells at the following time points: 24 h, 48 h, and 72 h after removing platelets. After incubation for 2 h, the absorbance was measured at a wavelength of 450 nm.

### Cell apoptosis

The Annexin V–FITC/propidium iodide (PI) staining kit (Invitrogen) was used to analysis the apoptosis of HT29 or Caco-2 cells by flow cytometry according the manufacturer’s instructions. For induced apoptosis of HT29 or Caco-2, the cells incubated with platelets were cultured in 24-well plates without fetal bovine serum. After washes with phosphate buffeted saline (PBS), the cells were resuspended in binding buffer (100 mM HEPES, 100 mM NaCl, and 25 mM CaCl_2_ [pH 7.4]) and stained with Annexin V-FITC/PI at room temperature in darkness for 30 min. After the cells were washed with PBS three times, an FACSCalibur (BD Biosciences) was used to evaluate the apoptosis ration by gating PI and Annexin V–positive cells. All experiments were performed in triplicate.

### In vivo tumor model

Six-week-old male BALB/c nude mice (Model Animal Research Center of Nanjing University, Nanjing, China) were used to establish the orthotopic transplantation model of human CRC. HT29 cells were subcutaneously injected into mice (1 × 105 cells in 0.2 mL PBS per mouse, 3 mice per group). The needle was inserted into the left side of the armpit, midway down, 5 mm deep at a 45° angle. After 10 days, 1 × 1010 platelets from CRC patients or HVs were injected into the same place every 2 days for 6 times. Mice were sacrificed at 24 days and the xenografted tumors were removed to measure the weights. The tumor tissues were used for protein and total RNA extraction for western blotting and qRT-PCR assay.

### Statistical analysis

Statistical analysis was performed with SPSS 16.0 software. Student’s *t*-test or two-sided χ^2^ test was used to compare the differences in other variables among the groups. A *P* value < 0.05 was considered to be statistically significant. ROC analysis was performed to estimate the diagnostic value of platelet mRNAs.

### Ethical approval

This study was approved by the Ethics Committee of Jiangsu Institute of Cancer Research, and written informed consent was obtained from all patients. All experiments were performed in accordance with relevant guidelines and regulations.

## Supplementary Material

Supplementary Figures

Supplementary Table 1
