# Supplementary material for: TIMP1 mRNA in tumor-educated platelets is diagnostic biomarker for colorectal cancer
Source: Aging (Albany NY). 2019 Oct 22;11(20):8998–9012. doi: 10.18632/aging.102366 (PMC6834400; doi:10.18632/aging.102366)
Supplement: Supplementary Figures [file aging-11-102366-s002.pdf]

SUPPLEMENTARY FIGURES

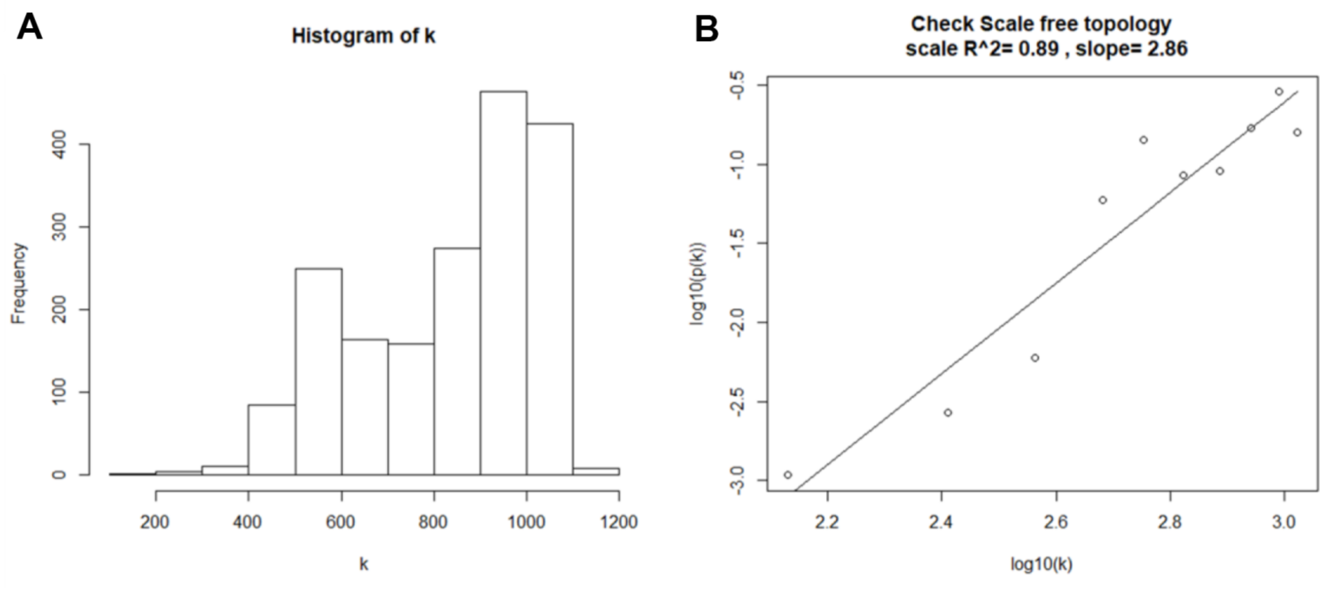

Supplementary Figure 1. Scale free topology when β = 1.

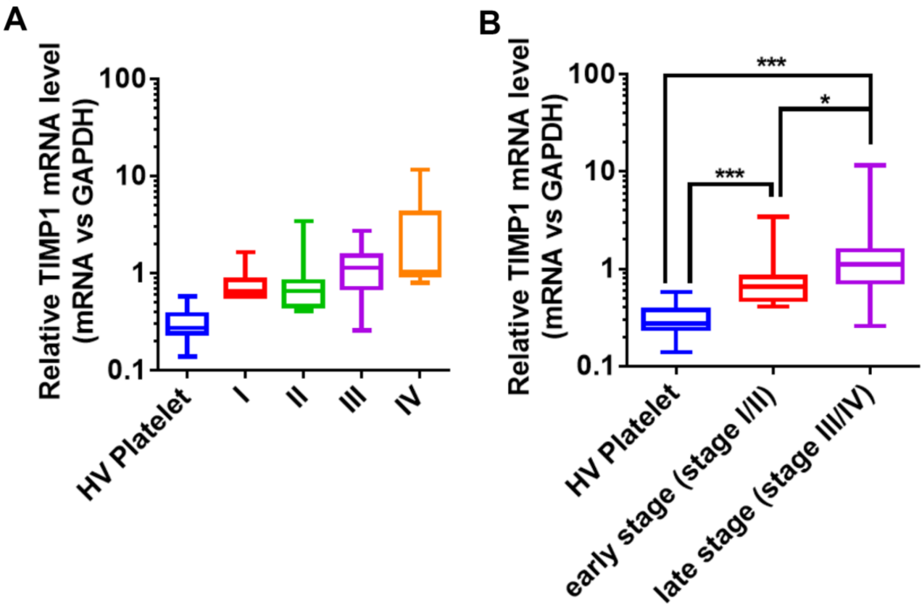

Supplementary Figure 2. The relative levels of TIMP1 mRNAs in the platelets from 286 CRC patients with different stages and 41 healthy volunteers by qRT- PCR (A-B). ***P < 0.001.

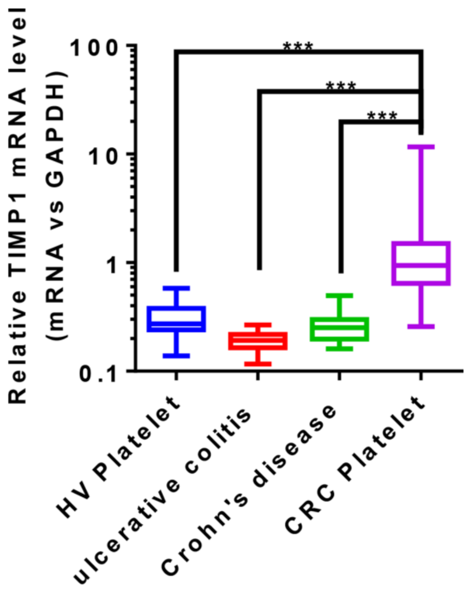

Supplementary Figure 3. The relative levels of TIMP1 mRNAs in the platelets from 286 CRC patients, 22 patients with ulcerative colitis, 23 patients with Crohn's disease and 41 healthy volunteers by qRT- PCR. ***P < 0.001.

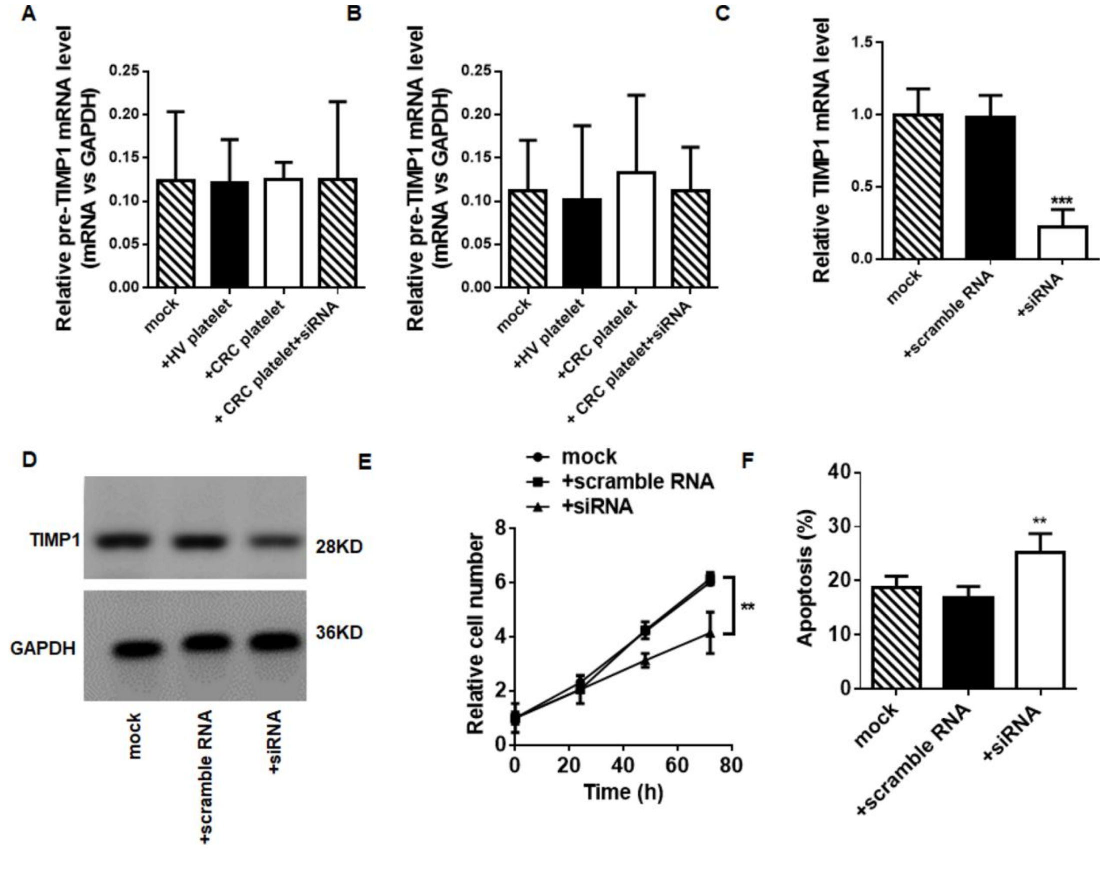

Supplementary Figure 4. The efficiencies of siRNA of TIMP1. (**A**–**B**) The pre-mRNA of TIMP1 in HT29 cells (**C**) and Caco2 cells (**D**) incubated with HV platelets and CRC platelets. (**C**–**D**) The mRNA and protein level of TIMP1 in HT29 cells transfected with scramble RNAs and siRNAs. (**E**–**F**) The proliferation (**E**) and apoptosis (**F**) assay of HT29 cells transfected with scramble RNAs and siRNAs. *p < 0.01, **p < 0.001.
