# Supplementary material for: TIMP1 mRNA in tumor-educated platelets is diagnostic biomarker for colorectal cancer
Source: Aging (Albany NY). 2019 Oct 22;11(20):8998–9012. doi: 10.18632/aging.102366 (PMC6834400; doi:10.18632/aging.102366)
Supplement: Supplementary Table 1 [file aging-11-102366-s001.pdf]

SUPPLEMENTARY TABLE

Supplementary Table 1. The primers used for qRT-PCR.

Gene	Forward	Reverse
ASAH1	AGATGTCATGTGGATAGGGTTCC	GGGGCCAATATCTTGGTCTTG
C12orf76	CTCGCTTCATTCATACCACAGT	CAGGGAGGATGTGCCTTTCA
FRMD3	ATGAAAACGCCTTTTTGATTGCT	AGTGGGTTCACCCGAATATGG
SVIP	ATGGGGCTGTGTTTTCCTTGT	TGTCCACCTAAGTCCACCTTC
ADIPOR1	ACGTTGGAGGGTCATCCCATA	AAACAGCACGAAACCAAGCAG
TIMP1	AGAGTGTCTGCGGATACTTCC	CCAACAGTGTAGGTCTTGGTG
RAB4A	GTCCGTGACGAGAAGTTATTACC	TGAGCGCACTTGTTTCCAAAA
ISCU	GGGTCCCTTGACAAGACATCT	CCTTTCACCCATTCAGTGGCTA
PGRMC1	AAAGGCCGCAAATTCTACGG	CCCAGTCACTCAGAGTCTCCT
CALM3	GACCATTGACTTCCCGGAGTT	GATGTAGCCATTCCCATCCTTG
